# Evaluating appropriate use of nictoine replacement therapy on acute adult psychiatric units and adverse events related to smoking bans on wards

**DOI:** 10.1192/bjo.2021.855

**Published:** 2021-06-18

**Authors:** Sabrina Hasnaoui, Virupa Ramachandran

**Affiliations:** Birmingham and Solihull Mental Health NHS Foundation Trust

## Abstract

**Aims:**

To assess if patients are offered appropriate and adequate NRT (nicotine replacment therapy) upon admission to an acute adult inpatient ward.

To evaluate the number of adverse events occurring which are related to mandatory temporary abstinence from cigarette smoking on inpatient wards.

**Background:**

Not all patients are able or willing to quit smoking when admitted to secondary care. In line with NICE Guidance, psychiatric inpatients should not be permitted to smoke inside the hospital building or outside on hospital grounds. NRT is the most widely used smoking cessation aid. It aims to temporarily replace the nicotine from cigarettes to reduce motivation to smoke and nicotine withdrawal symptoms.. NRT should be offered to patients who need support with nicotine withdrawal during an inpatient stay. Trust guidelines state nursing staff are able to administer NRT to patients on admission without prescription, reducing cravings and withdrawal symptoms such as agitation and anxiety which can lead to adverse events such as aggressive behaviour. Cravings may result in the patients self-discharging or absconding from the ward to smoke.

**Method:**

A retrospective review of electronic records was conducted of all inpatients admitted to two acute adult units over a three month period (65 patients). Patients were identified as smokers or non-smokers. Search words used included: ‘smoking’, ‘NRT’, ‘nicotine’, ‘cig’ to search for relevant entries. Data collected included whether NRT was offered and given by nursing staff on admission and adverse events related to smoking.

**Result:**

Data from 65 patient admissions were reviewed (31 males, 34 females, mean age 37 years). 37 (57%) patients were identified as smokers. NRT was offered and accepted by 17% of patients on admission and not recorded in 77% of admissions. NRT was declined when offered by 3% of patients.

Adverse events related to smoking occurred in 38% of ‘smoking’ patients. 40% of these adverse events occurred in first 72 hours of admission. Adverse events include verbal conflicts, physical aggression towards nursing staff and smoking in patient areas.

**Conclusion:**

The majority of patients were not offered NRT on admission or this was not accurately documented in clinical notes. When offered NRT, a large proportion of patients accepted it demonstrating its acceptability amongst this patient group. There is a high rate of adverse events related to smoking on wards. More accurate documentation is required to ensure NRT is being sufficiently offered to patients to reduce possible withdrawal symptoms and adverse events.

